# *AVPR1A* and *SLC6A4* Polymorphisms in Choral Singers and Non-Musicians: A Gene Association Study

**DOI:** 10.1371/journal.pone.0031763

**Published:** 2012-02-22

**Authors:** Andrew P. Morley, Madan Narayanan, Rebecca Mines, Ashraf Molokhia, Sebastian Baxter, Gavin Craig, Cathryn M. Lewis, Ian Craig

**Affiliations:** 1 King's Health Partners Academic Health Sciences Centre, London, United Kingdom; 2 Medical Research Council Social, Genetic & Developmental Psychiatry Centre, Institute of Psychiatry, King's College London, London, United Kingdom; 3 Division of Genetics and Molecular Medicine, King's College London, London, United Kingdom; University of Bristol, United Kingdom

## Abstract

Amateur choral singing is a common pastime and worthy of study, possibly conferring benefits to health and social behaviour. Participants might be expected to possess musical ability and share some behavioural characteristics. Polymorphisms in genes concerned with serotonergic neurotransmission are associated with both behaviour and musical aptitude. Those investigated previously include the variable number tandem repeats RS1, RS3 and AVR in the *AVPR1A* (arginine vasopressin receptor 1a) gene and STin2 in the *SLC6A4* (solute carrier family 6 [neurotransmitter transporter, serotonin], member 4) gene, as well as the *SLC6A4* promoter region polymorphism, 5-HTTLPR. We conducted a genetic association study on 523 participants to establish whether alleles at these polymorphisms occur more commonly in choral singers than in those not regularly participating in organised musical activity (non-musicians). We also analysed tagging single nucleotide polymorphisms (SNPs) for *AVPR1A* and *SLC6A4* to determine whether other variants in these genes were associated with singer/non-musician status. At the STin2 polymorphism, overall association with singer/non-musician status was evident at P = 0.006. The 9-repeat (P = 0.04) and 12-repeat (P = 0.04) alleles were more common in singers and the 10-repeat allele less so (P = 0.009). Odds ratios were 0.73 (95% CI 0.57–0.94) for the 10-repeat allele and 2.47 (95% CI 0.88–6.94) for the rarer 9-repeat allele. No overall association was detected at P<0.05 between any other polymorphism and singer/non-musician status. Our null findings with respect to RS3, RS1 and AVR, polymorphisms associated with musical ability by other authors, suggest that choir membership may depend partly on factors other than musical ability. In a related musical project involving one participating choir, a new 40-part unaccompanied choral work, “Allele”, was composed and broadcast on national radio. In the piece, each singer's part incorporated their personal RS3 genotype.

## Introduction

Choral singing is a common pastime which merits serious study. Singers in amateur choirs subjectively perceive physical, emotional, social and spiritual benefits [1]. Teenage American students involved in choirs or other non-sporting extracurricular activities have lower odds for alcohol use, binge drinking, marijuana use and vandalism than their counterparts whose activities are solely sport-related [2]. There is also evidence that singing may have biochemical effects. After singing, but not after listening to music, both positive affect and salivary secretory immunoglobulin A (S-IgA) are increased in choral singers [3]. In other contexts, S-IgA increases with relaxation [4] and decreases with stressful events [5].

Choral singing is a highly sociable activity. The genetics of social behaviour have long been the subject of extensive research and it now appears that some of the relevant genes and polymorphisms may influence musical ability as well. In a recent study, 298 individuals from 19 families were genotyped at pre-selected polymorphisms in five gene regions – *AVPR1A*, *SLC6A4*, *TPH1*, *COMT* and *DRD2*. All five genes code for proteins concerned with neurotransmission. The first three of these are involved in, or influenced by, the serotonergic system. Association was sought with the Karma Music Test and with the Seashore measures of musical perception, both formal tests of musical ability.

The associations with smallest P values were found between music test scores and *AVPR1A* haplotypes incorporating combinations of the highly variable promoter region VNTRs RS1, RS3 and the intronic AVR. A weak association was also detected between the Karma Music Test score and an *SLC6A4* haplotype combining the intronic VNTR, STin2, and the promoter region 5-HTTLPR [6]. In another study, RS1 and RS3 polymorphisms also influenced the likelihood of an individual's involvement in creative dance - an association strengthened when conditional on polymorphisms in the regulatory regions of *SLC6A4* [7].

The *AVPR1A* RS3 polymorphism has been studied for its associations with social, behavioural and personality traits [8]. Its length appears to influence pair-bonding [9] and altruism [10]. Polymorphisms in the *SLC6A4* gene have been the subject of sustained interest in psychiatry, in particular the 5-HTTLPR polymorphism. This has been associated with depression [11], autism [12], obsessive compulsive disorder [13] and antidepressant response [14].

Given that *AVPR1A* and *SLC6A4* polymorphisms apparently influence both social and musical traits, we believed choral singers represented a group which might usefully be studied further. Our hypothesis was that allelic variants at complex polymorphisms in *AVPR1A* and *SLC6A4*, associated with musical phenotypes by Ukkola et al. [6] and Bachner-Melman et al. [7], would be more common in choral singers than in non-musicians. We aimed to determine this with a gene association study. To capture genetic variability more fully than the earlier studies, we also compared genotypes between the two groups at a set of tagging polymorphisms for *AVPR1A* and *SLC6A4*.

## Methods

### Ethics statement

This study was approved by St. Thomas' Research Ethics Committee and written informed consent was obtained from all participants.

### Data collection

We recruited 523 individuals, aged 16–90 years and of white ethnicity. Potential participants determined their ethnic eligibility by referring to our institution's diversity monitoring list. This includes 70 individual designations in five broader categories – “White”, “Black or black British”, “Asian or Asian British”, “Mixed background” or “Other ethnic groups”. Self identified ethnicity correlates well with ancient geographic ancestry, a major determinant of genetic structure [15].

The choral group (n = 262) comprised regular singers from nine different amateur choirs. All choirs required singers to audition for membership and were deemed by one of the authors (AM) to be of a moderate to high standard on the basis of repertoire, performance history and reputation. The non-musician group (n = 261) was recruited from among staff at Guy's and St. Thomas' Hospitals and the MRC Social, Genetic and Developmental Psychiatry Centre, as well as patients attending for outpatient surgery and their relatives. Additional participants in the non-musician group were recruited from doctors attending academic meetings in London and the south of England. Each participant in the non-musician group was required to confirm that they did not “participate regularly in any organised musical activity, including a choir, dance class, orchestra or rock band”.

For each participant recruited to the study, gender, date of birth and musical status (choral singer or non-musician) was recorded. Each participant provided cheek swabs for DNA analysis.

### Genotyping

For *SLC6A4* and its 1000 bp margins, we genotyped the same panel of polymorphisms as tested in a recent paper by our group [16] with one omission. The panel included a set of tagging SNPs, selected using the SNPTagger program (www.broad.mit.edu/mpg/tagger) run in Windows XP and the HAPMAP data on the CEPH CEU population with European ancestry (CEPH NCBI Build 35/UCSC hg17/May 2004 coordinates) [17]. Selection criteria were a minimal allele frequency of 5% in the white population and pairwise r^2^ = 0.8. The ten selected SNPs provided 92% coverage of the DNA sequence variation in the *SLC6A4* gene. In the current study, as before, we genotyped the *SLC6A4* promoter region 5-HTTLPR and the VNTR STin2 (intron 2). We omitted the VNTR StIn4 (intron 4), which we previously found to be the only other polymorphic *SLC6A4* VNTR. This is because our earlier study showed it to be in tight linkage disequilibrium with neighbouring markers [16].

For the current study, we also applied the same tagging criteria to *AVPR1A* and its 1000 bp margins, generating a set of three SNPs. We also genotyped those *AVPR1A* repeat polymorphisms investigated by others in the context of musical phenotypes [6], [7], namely RS1, RS3 and AVR.

DNA was extracted from buccal swabs provided by participants as previously described [18]. SNPs in the *AVPR1A* and *SLC6A4* genes were determined using Taqman SNP Genotyping Assays (Applied Biosystems) and analysed on a 7900HT Sequence Detection System (Applied Biosystems). The 5-HTTLPR and the rs25531 SNP were genotyped together using a two-stage method described previously [16]. The regions containing the other VNTRs were amplified by PCR using the following flanking oligonucleotide primer pairs: Stin2 forward 5′-FAM-GTCAGTATCACAGGCTGCGAG-3′ and reverse 5′-GTTTCTTTGTTCCTAGTCTTACGCCAGTG-3′; RS1 forward 5′-FAM-AGGGACTGGTTCTACAATCTG-3′ and reverse 5′-GTTTCTTACCTCTCAAGTTATGTT GGTG-3′; RS3 forward 5′-FAM-CCTGTAGAGATGTAAGTGCT-3′ and reverse 5′-GTTTCTTTCTGGAAGAGACTTAGATGG-3′; AVR forward 5′-FAM-ATCCCATGTCCGTCTGGAC-3′ and reverse 5′-FAM-ATCCCATGTCCGTCTGGAC-3′. The product sizes were determined by running on a 3130xl Genetic Analyzer (Applied Biosystems).

### Statistical analysis

Statistical analysis was performed using UNPHASED [19] to test for association between choral singer/control status and SNPs, VNTRs and 5-HTTLPR. PLINK was used for quality control for SNPs, assessing completeness of genotyping, allele frequency and departures from Hardy-Weinberg equilibrium [20]. Testing for relatedness between study participants was not possible with the limited genotyping performed. Markers were analysed as single polymorphisms and in two-locus haplotypes within each gene. Across genes, specific tests of association of *AVPR1A* polymorphisms and two-locus haplotypes with singer/non-musician status, conditional on the 5-HTTLPR and STin2 VNTRs, were performed in an attempt to replicate previous findings [7]. At each VNTR, rare alleles (<5% frequency) were pooled with alleles of similar length but higher frequency to increase power to detect association (Results section, table 1). This pragmatic strategy is standard in analysing highly polymorphic VNTRs and predicated on the hypothesis that alleles with similar number of repeats at a given VNTR have common ancestry.

**Table 1:** Association of *AVPR1A* and *SLC6A4* VNTRs with singer/non-musician status.

VNTR/pooled alleles or repeats	Individual alleles	Choral singers (n = 261) Number (%) of alleles	Non-musicians (n = 258) Number (%) of alleles	Odds ratio (95% CI)	P value*
***AVPR1A*** ** RS3**					
1	320, 327,329, 331	47 (9)	35 (7)	1.35 (0.82–2.23)	0.20
2	333	40 (8)	43 (8)	0.94 (0.57–1.54)	0.65
3	335	128 (25)	129 (25)	1	0.77
4	337	128 (25)	113 (22)	1.14 (0.80–1.62)	0.37
5	339	39 (7)	57 (11)	0.69 (0.43–1.11)	**0.04**
6	341, 343	82 (16)	79 (15)	1.05 (0.71–1.55)	0.92
7	345,347,349,350,352	54 (10)	50 (10)	1.09 (0.08–0.77)	0.77
				Overall significance	0.39
***AVPR1A*** ** RS1**					
1	309,313	64 (12)	72 (14)	0.88 (0.60–1.30)	0.43
2	317	215 (41)	214 (41)	1	0.97
3	321	120 (23)	128 (25)	0.93 (0.68–1.28)	0.51
4	325	44 (8)	39 (8)	1.12 (0.70–1.80)	0.59
5	329, 333, 337, 341, 345	77 (15)	63 (12)	1.22 (0.83–1.78)	0.22
				Overall significance	0.66
***AVPR1A*** ** AVR**					
1	211, 214, 215, 217	86 (17)	82 (16)	1.05 (0.74–1.50)	0.76
2	218	61 (12)	56 (11)	1.09 (0.73–1.63)	0.64
3	219	75 (15)	75 (15)	1.00 (0.70–1.44)	0.98
4	221	255 (49)	256 (50)	1	0.90
5	223, 225, 226, 227	39 (8)	45 (9)	0.87 (0.55–1.38)	0.48
				Overall significance	0.95
***SLC6A4*** ** STin2**					
9		14 (3)	5 (1)	2.47 (0.88–6.94)	**0.04**
10		176 (34)	212 (41)	0.73 (0.57–0.94)	**0.009**
12		330 (63)	291 (56)	1	**0.04**
				Overall significance	**0.006**

*The P value for each allele is derived from the test of association between choral singers/controls for that allele against all other alleles pooled.

In this exploratory study, we avoided the highly conservative Bonferroni correction [21], instead correcting for multiple testing within genes with SNPSpD. This method uses linkage disequilibrium between a set of SNPs to determine the number of independent tests [22]. For *SLC6A4*, 10 genotyped SNPs were equivalent to 5 independent tests. Adding the 2 VNTRs genotyped gives 7 tests and a p-value threshold for gene-wide significance of 0.05/7, or 0.0071. For *AVPR1A*, 3 SNPs contribute two independent tests. When these are combined with the 3 VNTRs, the p-value threshold for gene-wide significance is 0.05/5 = 0.01. Only those P values reaching these gene-wide thresholds can be interpreted as statistically significant. No correction for multiple testing across genes has been applied.

Power calculations [23] indicated that a sample size of 250 choral singers and 250 controls would provide power of 78% to detect a difference in allele frequency assuming each allele confers a two-fold increased chance of being a choral singer, for an allele frequency of 0.05 (at a significance level of 0.05). With a higher allele frequency of 0.1, power is 96%. At 0.2, power rises to 99% for the same two-fold increase, and is sufficient, at 84%, to detect a much lower odds ratio – 1.5 and 2.25 for hetero- and homozygotes respectively. A sample size of 500 also means that genotyping an SNP with an allele frequency of 0.1 in LD (r^2^ = 0.9) with the causal variant provides power of 92%. The range of theoretical allele frequencies in these calculations are broadly in keeping with actual frequencies previously reported for our genotyped polymorphisms [6], [7], [16]. The calculations suggest that the study was sufficiently well-powered to detect association in the proposed sample size, across a wide range of marker properties.

## Results

After extraction, adequate DNA samples were available for 261 singers and 258 non-musicians. Of these participants, three failed to state full age and gender information. From the available data, mean age (SD) of the singers was 42 (15) years and 122∶139 (47%∶53%) were male∶female. Corresponding figures for the non-musicians were 40 (12) years and 117∶139 (45∶54%). Genotyping failure did not exceed 2% at any polymorphism except 5-HTTLPR (19%). For *AVPR1A* and *SLC6A4*, the designated gene-wide P value thresholds for significance were 0.01 and 0.0071 respectively (see Methods).

### *AVPR1A*

**Figure 1:**
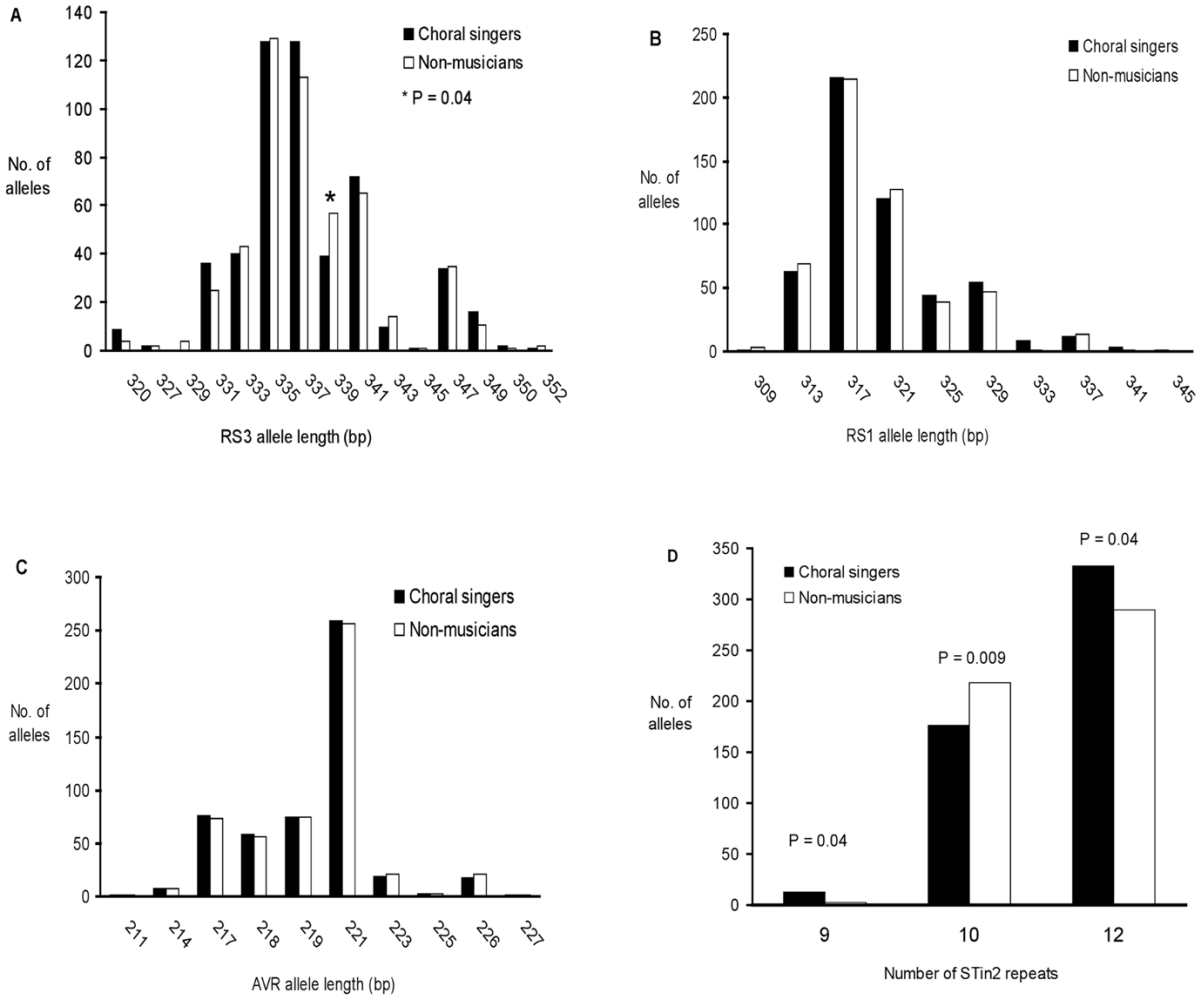
Allele frequencies in choral singers (n = 261) and non musicians (n = 258) for *AVPR1A* and *SLC6A4* VNTRs. (A) RS3 (B) RS1 (C) AVR and (D) STin2.

Allele frequencies at the RS3, RS1 and AVR VNTRs are illustrated in figure 1. Having pooled alleles of frequency <5%, no significant overall association of any VNTR with musical status was seen. Pooled allele frequencies for *AVPR1A* VNTRs, with estimates of individual allelic effects, are given in table 1. No significant effect of carrier status was observed for any allele. None of the VNTRs demonstrated a significant effect in logistic regression models incorporating a sex/musical status interaction term.

All *AVPR1A* SNPs were in Hardy-Weinberg equilibrium (HWE). No SNP was significantly associated with musical status (table 2) and this finding was unaffected by the introduction of age in a logistic regression model.

**Table 2:** Association of *AVPR1A* and *SLC6A4* SNPs, and 5 -HTTLPR, with singer/non-musician status.

Gene/polymorphism	Location (basepairs)	Individual alleles (minor/major)	Minor allele frequency in choral singers (n = 261)	Minor allele frequency in non-musicians (n = 258)	Odds ratio (95% CI)	P value
***AVPR1A***						
rs1042615	63544209	A/G	0.42	0.42	1.01 (0.79–1.29)	0.93
rs10877969	63547239	C/T	0.14	0.14	1.05 (0.74–1.50)	0.78
rs11174811	63540476	A/C	0.14	0.15	0.94 (0.67–1.33)	0.73
***SLC6A4***						
5-HTTLPR	28564498+	(La+Lg)/(Sa+Sg)*	0.42	0.40	1.10 (0.83–1.45)	0.50
rs2020933	28561755	T/A	0.05	0.06	0.81 (0.47–1.41)	0.46
rs2066713	28551665	A/G	0.37	0.42	0.81 (0.63–1.04)	0.09
rs2020939	28550732	A/G	0.44	0.39	1.24 (0.97–1.59)	0.08
rs8076005	28547210	G/A	0.20	0.20	1.02 (0.75–1.39)	0.89
rs2020942	28546914	T/C	0.37	0.42	0.81 (0.63–1.04)	0.11
rs140700	28543389	T/C	0.10	0.10	1.02 (0.69–1.53)	0.90
rs4583306	28538715	G/A	0.42	0.38	1.18 (0.92–1.51)	0.19
rs140701	28538532	T/C	0.42	0.38	1.19 (0.93–1.52)	0.17
rs4325622	28526475	C/T	0.47	0.43	1.15 (0.90–1.47)	0.25
rs3813034	28524804	C/A	0.46	0.43	1.14 (0.90–1.46)	0.28

*At 5-HTTLPR long alleles La and Lg have been combined, as have short alleles Sa and Sg, to permit analysis as a bi-allelic marker.

Two-marker *AVPR1A* haplotypes, incorporating SNPs, VNTRs or one of each, showed no significant overall association with musical status, the lowest P value being 0.018 (for both RS3-rs2066713 and RS3-2020942 haplotypes).

### *SLC6A4*

The rs25531 SNP (A/G) is contained within 5-HTTLPR (long/short) so that the combined assay used to characterise the region identifies four alleles; La, Lg, Sa, Sg. In our study, frequencies were low for Sg (0.2%) and Lg (6.6%). Consequently, statistical analysis was conducted only with respect to allele length, pooling La with Lg, and Sa with Sg to create a bi-allelic marker (L/S). Allele length was not associated with musical status (table 2).

The STin2 VNTR allele frequencies differed between the two groups. These, and the estimates of allele effects, are illustrated in figure 1 and table 1 respectively. There was an overall association with singer/non-musician status at this locus (P = 0.006). The 9-repeat (STin2.9, P = 0.04) and 12-repeat (STin2.12, P = 0.04) alleles were more common in choral singers and the 10-repeat allele (STin2.10) more common in non-musicians (P = 0.009). Odds ratios were 0.73 (95% CI 0.57–0.94) for the 10-repeat allele and 2.47 (95% CI 0.88–6.94) for the rarer 9-repeat allele compared to the baseline 12-repeat allele. For no haplotype incorporating STin2 and another locus, whether SNP or VNTR, did the additional locus decrease the P value for association below that of STin2 alone.

All ten *SLC6A4* SNPs were in HWE in the non-musician group, though three deviated from HWE in the choral singer group (rs3813034, P = 0.025; rs4325622, P = 0.046; rs2020939, P = 0.024). None were significantly associated with musical status, either alone (table 2) or with age in a logistic regression model. Of all possible two-locus haplotypes within the *SLC6A4* gene, incorporating SNPs alone or with 5-HTTLPR, none were significantly associated with musical status, the lowest P value being 0.038 for rs2020933-rs8076005.

### Conditional associations

Of all *AVPR1A* markers and two-locus haplotypes, none showed an overall association with singer/non-musician status whether conditional on 5-HTTLPR or STin2, the lowest P value being 0.03 for RS1-RS3 conditional on 5-HTTLPR.

## Discussion

Our hypothesis in this study was that allelic variants in *SLC6A4* and *AVPR1A* genes, known to be associated with musical aptitude, would also be associated with a related behavioural outcome, namely choir membership. *A priori* power calculations indicated that our sample size would be adequate to detect effects on this outcome of our chosen polymorphisms, assuming broadly similar allele frequencies in our participants to those reported by other groups. This assumption proved correct. After statistical correction for multiple testing, we found that STin2.9 and STin2.12 alleles, in the *SLC6A4* gene, were more common in choral singers than in non-musicians, and the STin2.10 allele less common.

In the same two groups, no overall difference was detected for allele frequencies in the *AVPR1A* gene at RSI, RS3 and AVR polymorphisms. These *AVPR1A* polymorphisms have been associated with performance in musical tests in a study which, to our knowledge, is the only one other than our own to address a possible association between STin2 and phenotypes related to music. In that study, Karma Music Test scores were associated with the haplotype 5-HTTLPR.La/STin2.12 [6].

Our allele frequencies for STin2 VNTR, a repeating 17-bp motif, were broadly in keeping with those in similar populations, with STin2.12 and STin2.10 being common alleles and STin2.9 a rare allele. We also found a single 11-repeat allele, a finding consistent with other studies [24]. We found no differences between groups in allele frequencies at 5-HTTLPR, the other *SLC6A4* VNTR we studied. At 19%, the genotyping failure rate at this polymorphism was high but this may reflect the complex nature of the assay, including both the repeat and associated SNP.

Validation of our findings will require replication in an independent sample. Furthermore, while we attempted to select a study population of reasonable ethnic homogeneity, we made no correction for cryptic population structure in the association analysis. Failure to apply such correction is believed to be a factor contributing to false positive results in candidate gene studies like our own [25]. It was not feasible for us to conduct the additional genotyping typically employed for correction purposes in genome-wide association studies. Our results, then, must be regarded as preliminary ones that must be tested more robustly with the appropriate controls for population structure.

STin2 has been widely investigated in connection with psychiatric or neurological disease. Conflicting studies report associations between all three major STin2 alleles and depression-related phenotypes [26], [27], [28], [29]. Findings relating to psychotic symptoms in Alzheimer's patients are similarly inconsistent, with some studies implicating STin2.12 [30] and others STin2.10 [31]. STin2 alleles may [32] or may not [33] be associated with obsessive-compulsive disorders. The STin2.12 homozygote genotype has been associated with increased risk of schizophrenia [34], post stroke depression [35], migraine [36], [37], [38], a better response to drug treatment in depression [39], [40] and premature ejaculation [41] but a more inconsistent one to triptan therapy in migraine [42]. Haplotypes incorporating STin2 and the *SLC6A4* 5-HTTLPR polymorphism have been associated with sleep apnoea [43], postpartum depression [44] and attention deficit hyperactivity disorder [45].

The literature hints at some causative mechanisms behind these statistical associations. In a variety of experimental contexts, Stin2 appears to affect transcriptional regulation, the STin2.12 allele enhancing gene expression more than Stin2.10 [46], [47], [48]. Some haplotypes combining STin2 and 5-HTTLPR alleles also appear to influence transcriptional regulation by the transcription factor, CCTC-binding factor [49].

Relationships between these in vitro phenomena and clinical biochemical effects are also suggested by recent studies. In autistic individuals, for example, the STin2.10 allele is found more commonly in those with hyperserotonemia than in those with normal 5-HT [50]. Among male Han Chinese patients with obstructive sleep apnoea syndrome, those with a specific haplotype incorporating STin2.12 have lower plasma 5-HT level and 5-hydroxyindolacetic acid (5-HIAA) levels compared with non- STin2.12 carriers [43]. 5-HT availability, assessed by single photon emission computed tomography, is reduced in suicide attempters with a STin2.12 allele [51].

The influence of STin2 on personality in healthy individuals, such as the participants in this study, has also been investigated. There is some evidence of an association between the polymorphism and reward dependence or cooperation [52]. Individuals with the STin2.10 allele appear to be more risk tolerant to financial losses than those with the Stin2.12 allele [53]. The STin2.10 allele is also associated with lower neuroticism scores, as measured by the Temperament and Character Inventory, and lower harm avoidance scores in the Eysenck Personality Inventory [54].

Choir membership represents a complex behavioural phenotype and the two genes we investigated affect both musical ability and behaviour. As a single response from each participant in our study determined phenotype designation, “choir member” or “non-musician”, we cannot say whether the difference in allele frequencies between the groups reflects STin2 effects on musical ability or on some other behavioural trait.

Regarding musical ability, one would expect choral singers to share many genetic characteristics with those scoring highly in objective tests of musical aptitude. The fact that *AVPR1A* “musical genotypes”, identified with more certainty than STin2 by other authors, were similarly distributed in our choral singers and non-musicians may relate to our phenotype selection. Practical considerations mean that we were obliged to study amateur choral singers, albeit those at a very high level. Had we recruited professional singers for our choral singer group, they might have had a higher frequency of musical genotypes. Similarly, non-musicians identified themselves solely by reference to non-participation in musical activities. Some individuals in this group may merely have been deprived of relevant opportunities and were, in fact, musical but ignorant of the fact. If we had used formal music tests to select our two groups, the phenotypic distribution for musical ability in our study might have been closer to that of other studies.

As far as behavioural traits are concerned, membership of a successful choir entails many qualities in addition to, and perhaps for some individuals instead of, those assessed by formal musical tests. It is possible that these qualities are the ones associated with the STin2 polymorphism in our singers, rather than musical ability. Leaving aside a good voice, behavioural characteristics required of choir members include enthusiasm, concentration and attention to detail. A choral singer must also be able to modulate his/her own voice in response to the overall sound of the choir and to understand non-verbal cues from the conductor. The STin2 effect we observed may relate to one of these characteristics, rather than musical ability. If so, one might expect the STin2 allele frequency distribution to differ between choral singers and other musicians for whom some of these skills are arguably less important, such as conductors or solo instrumentalists.

Social behaviour that is not choir specific may also account for the STin2 effect we observed. The polymorphism may contribute to a “predisposed to group activity” phenotype. Our non-musicians were recruited individually at one-off educational meetings, where delegates could not be said to represent an ongoing social unit like a choir. In this respect, it might have been instructive to study STin2 allele frequencies in a third cohort comprising individuals in another established social group unrelated to music - cricket club members, for example.

Finally, the effect we identified might relate to behavioural traits in the non-musician group and not the choral singers. While very few of our singers were hospital workers, the majority of subjects in our non-musician group were. Of these many were doctors, particularly anaesthetists. It is possible that STin2-related personality traits are more common among hospital staff than those employed elsewhere.

This study was conducted in conjunction with a musical project involving one participating choir. A new 40-part unaccompanied choral work, “Allele”, was composed and broadcast on national radio. In the piece, each singer's part incorporated their personal RS3 genotype. Further details are provided in Text S1.

In conclusion, the STin2 VNTR in the *SLC6A4* gene is associated with choir membership in this study. Allele frequencies at other *AVPR1A* and *SLC6A4* polymorphisms, more strongly associated with musical phenotypes than STin2 by other authors [6], were similar in choral singers and non-musicians. Our study therefore failed to confirm the hypothesis that these polymorphisms are associated with musical ability, as assessed by choir membership. One possible interpretation of our results is that genetic factors other than those affecting musical ability alone may influence whether an individual belongs to a choir or not.

## Supporting Information

Text S1 **The composition and performance of “Allele”.**
(DOC)
