# Supplementary material for: AVPR1A and SLC6A4 Polymorphisms in Choral Singers and Non-Musicians: A Gene Association Study
Source: PLoS One. 2012 Feb 22;7(2):e31763. doi: 10.1371/journal.pone.0031763 (PMC3285181; doi:10.1371/journal.pone.0031763)
Supplement: Text S1 — The composition and performance of “Allele”. (DOC) [file pone.0031763.s001.doc]

Forty choral singers from the New London Chamber Choir, who participated in the gene association study, also took part in a musical experiment. This entailed the composition and performance of a new unaccompanied choral work. The text, "Allele" by Ruth Padel, was specially commissioned. Each individual singer consented to the anonymous release of their RS3 genotyping results, expressed as two allele lengths (bp), to the composer Michael Zev Gordon. Of the 18 polymorphisms genotyped, a VNTR was selected in preference to a SNP because the larger number of alleles was deemed likely to lead to a more interesting choral work. As the composition of the piece was started before the results of the gene association study were known, the VNTR chosen was RS3. At the outset of the study, it was thought to be the VNTR that would most likely to be associated with choir membership.

For each pair of allele lengths, the composer was told the corresponding vocal part normally sung by that individual. He was also given the two primer sequences for the RS3 analysis. These sequences were used directly in musical motifs in the piece, with the musical notes A, C and G corresponding to the relevant bases and thymine (T) appearing as the note B, as in the do-re-mi system of musical notation.

Three hundred was subtracted from each allele length, leaving two numbers between 19 and 41 for every singer. These were used by the composer to determine the length in semiquavers of a critical musical phrase, personal to each singer, in an aleatoric section approximately two thirds of the way through the work. The score was written for eight sub-choirs of five singers, as in the famous 40 part renaissance work "Spem in Alium", by Thomas Tallis.

The world premiere of Allele by Michael Zev Gordon, sung by the New London Chamber Choir and directed by James Weeks, took place at the Diamond Light UK National Synchrotron Facility on 9th July 2010. It was broadcast on BBC Radio 3 on 25th September 2010.
